# Treatment of endothelial cell dysfunction in atherosclerosis: a new perspective integrating traditional and modern approaches

**DOI:** 10.3389/fphys.2025.1555118

**Published:** 2025-03-26

**Authors:** Luqun Yang, Xinjian Li, Lin Ni, Yuanyuan Lin

**Affiliations:** Third Hospital of Shanxi Medical University, Shanxi Bethune Hospital, Shanxi Academy of Medical Sciences, Tongji Shanxi Hospital, Taiyuan, China

**Keywords:** atherosclerosis, endothelial cell dysfunction, oxidative stress, inflammation, nanomaterials, stem cell therapy, genetic therapy

## Abstract

Atherosclerosis (AS), a prime causative factor in cardiovascular disease, originates from endothelial cell dysfunction (ECD). Comprising a vital part of the vascular endothelium, endothelial cells play a crucial role in maintaining vascular homeostasis, optimizing redox balance, and regulating inflammatory responses. More evidence shows that ECD not only serves as an early harbinger of AS but also exhibits a strong association with disease progression. In recent years, the treatment strategies for ECD have been continuously evolving, encompassing interventions ranging from lifestyle modifications to traditional pharmacotherapy aimed at reducing risk factors, which also have demonstrated the ability to improve endothelial cell function. Additionally, novel strategies such as promising biotherapy and gene therapy have drawn attention. These methods have demonstrated enormous potential and promising prospects in improving endothelial function and reversing AS. However, it is essential to remain cognizant that the current treatments still present significant challenges regarding therapeutic efficacy, long-term safety, and ethical issues. This article aims to provide a systematic review of these treatment methods, analyze the mechanisms and efficacy of various therapeutic strategies, with the goal of offering insights and guidance for clinical practice, and further advancing the prevention and treatment of cardiovascular diseases.

## 1 Introduction

Cardiovascular disease is one of the leading causes of death globally (Mensah et al., 2023; Martin et al., 2025). Atherosclerosis (AS), the pathological cornerstone of such diseases, is a chronic, systemic, progressing inflammatory condition (Hansson and Hermansson, 2011; Zhu et al., 2018; Waksman et al., 2024). The characteristic trait of this disease involves the accumulation of lipids and/or fibrous components in the endothelium, leading to arterial wall thickening, vascular luminal narrowing, and a decrease in vascular elasticity (Libby et al., 2019; Jebari-Benslaiman et al., 2022). As the disease progresses, plaques may rupture or erode, leading to thrombosis and even triggering life-threatening cardiovascular events (Asada et al., 2018; Smit et al., 2020; Tomaniak et al., 2020; Jebari-Benslaiman et al., 2022). Endothelial cells form a precise barrier between the blood vessel wall and blood, performing numerous essential functions (Deanfield et al., 2007; Cahill and Redmond, 2016; Pi et al., 2018; Krüger-Genge et al., 2019). Mounting evidence suggests that endothelial cell dysfunction (ECD) serves as an initial trigger and pivotal step in the development of AS (Gimbrone and García-Cardeña, 2016). However, this damage exhibits a certain degree of reversibility: upon elimination of pathological factors, the function of early-damaged endothelial cells may be restored (Poredos et al., 2021). In recent years, despite continuous innovation in therapeutic approaches targeting ECD in AS, such as the introduction of emerging technologies including nanotechnology, stem cell therapy, and genetic therapy, alongside lifestyle modifications and conventional pharmacological interventions, significant progress has been achieved to some extent. However, in implementing personalized treatment strategies and ensuring long-term efficacy and safety, we still face some unresolved challenges.

**FIGURE 1:**
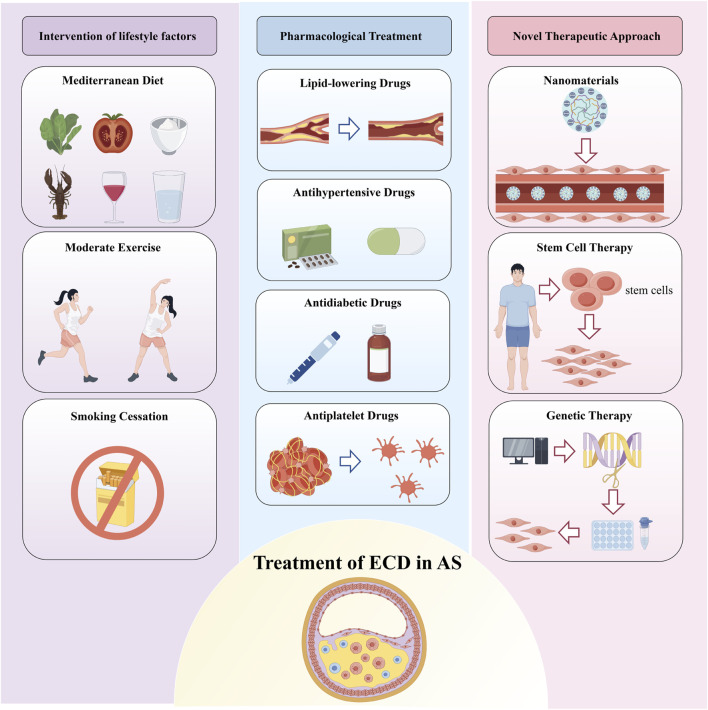
Treatment of Endothelial Cell Dysfunction in Atherosclerosis. Therapeutic strategies for endothelial cell dysfunction (ECD) in atherosclerosis (AS) are primarily categorized into three groups: lifestyle interventions, pharmacological treatments, and emerging therapies such as nanomedicine, stem cell therapy, and gene therapy. Lifestyle interventions include healthy dietary changes, regular physical activity, and smoking cessation. Pharmacological treatments refer to medications that not only reduce risk factors for AS but also improve endothelial function. These combined approaches aim to restore endothelial function and reduce the risk of developing AS.

Therefore, this review aims to summarize and analyze various therapeutic strategies for ECD in AS. We delve into the action mechanisms and potential applications of these methods, providing valuable insights for future scientific research and clinical practice (Figure 1).

## 2 Pathophysiology of AS

**FIGURE 2:**
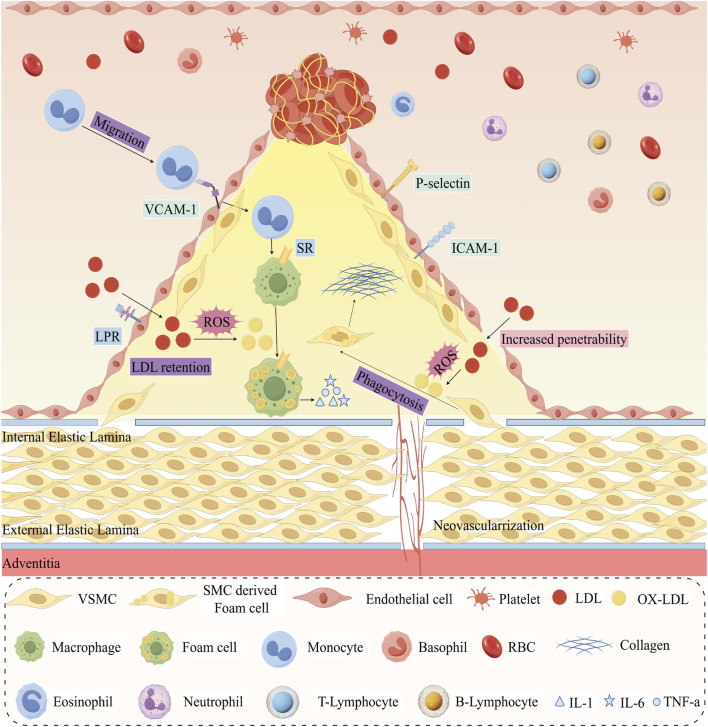
Pathophysiology of Atherosclerosis. VSMC: Vascular Smooth Muscle Cell; RBC: Red Blood Cell; LDL: Low-density lipoprotein; ox-LDL: oxidized LDL; VCAM-1: Vascular Cell Adhesion Molecule-1; ICAM-1: Intracellular Adhesion Molecule-1; IL-1: Interleukin 1; TNF-a: Tumor Necrosis Factor Alpha.

AS initiates with endothelial cell injury and dysfunction (Gimbrone and García-Cardeña, 2016; Berenji Ardestani et al., 2020). This damage escalates vascular permeability, facilitating positively charged lipoproteins to penetrate and accumulate in the negatively charged, proteoglycan-rich subendothelial region and undergo oxidative modification, setting the stage for atherosclerotic progression (Mundi et al., 2018; Lu et al., 2022; Qiao et al., 2022). At this phase, expression levels of Vascular cell adhesion molecule 1 (VCAM-1), Intercellular adhesion molecule 1 (ICAM-1), Monocyte chemoattractant protein-1 (MCP-1), P-selectin, and E-selectin are upregulated, guiding monocytes to transverse the endothelium (Zhu et al., 2018; Kong et al., 2022). Locally, under the action of Macrophage-colony stimulating factor (M-CSF), these monocytes transform into macrophages within the vessel wall, acquiring a pro-inflammatory or anti-inflammatory phenotype based on surrounding microenvironmental signals (Kim K. W. et al., 2020; Eshghjoo et al., 2022). Pro-inflammatory macrophages, through scavenger receptors (SRs) such as SR-a, CD36, and CD68, uptake oxidized low-density lipoproteins (ox-LDL), consequently transforming into foam cells filled with substantial cholesterol esters (Tabas and Bornfeldt, 2016; Chistiakov et al., 2017a; Chistiakov et al., 2017b). The appearance of foam cells may impair their original immune function and weaken their ability to clear dying cells (Schrijvers et al., 2005; Kojima et al., 2017). Furthermore, they can release Tumour Necrosis Factor-Alpha (TNF-a), Interleukin 1 (IL-1), IL-6, Interferon-gamma (IFN-γ), Platelet-derived Growth Factor (PDGF), MCP-1, CC Chemokine Receptor 2 (CCR2), CCR5, and other factors, assisting in recruiting more immune cells and thus intensifying intravascular inflammatory reactions. Apoptosis or necrosis of foam cells paves the way for the formation of a lipid core comprised of cholesterol esters, cellular debris, and cholesterol crystals, escalating rupture risks at lesion sites (Gui et al., 2022; Neels et al., 2023). Subsequently, vascular smooth muscle cells (VSMCs) transition to a proliferative state, migrating from the media to the intima and producing extracellular matrix components like collagen, elastin, and proteoglycans (Grootaert and Bennett, 2021). These processes contribute to the formation of a fibrous cap and thickening of the vessel wall, imparting stability to the plaque and resulting in luminal narrowing (Bennett et al., 2016; Watson et al., 2018). Thinning of fibrous caps coupled with the expansion of necrotic core zones may lead to plaque rupture, exposing prothrombotic materials, triggering platelet activation, thrombus formation, and ultimately local blood flow interruption or distal arterial embolism (Figure 2) (Shah et al., 1995; Bobryshev et al., 2008).

## 3 ECD

**FIGURE 3:**
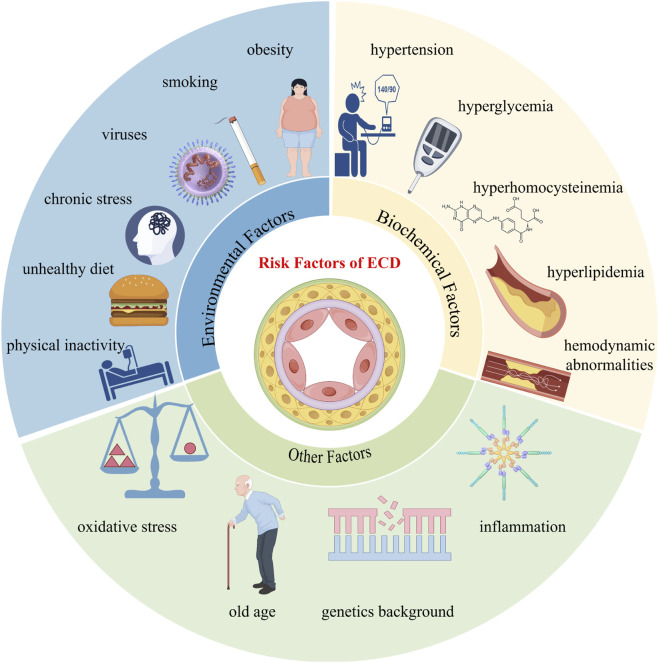
Risk factors of endothelial cell dysfunction.

ECD is defined as a series of maladaptive endothelial functional phenotypic changes associated with an increased risk of cardiovascular diseases (Gimbrone and García-Cardeña, 2016). Various environmental factors, including smoking, physical inactivity, unhealthy diet, chronic stress, obesity, viruses, and infectious microbes, as well as endogenous biochemical factors such as hypertension, hyperglycemia, hyperhomocysteinemia, hyperlipidemia, and local hemodynamic abnormalities, can all potentially trigger and exacerbate endothelial cell damage, leading to oxidative stress, further amplifying vascular inflammation, and driving the progression of AS (Figure 3) (Rajendran et al., 2013; Schrottmaier et al., 2020; Sher et al., 2020; Volino-Souza et al., 2020; Biering et al., 2021; Gallo et al., 2021; Yuan et al., 2022; Cheng H. et al., 2023; Shen et al., 2023; Ishida et al., 2024; Naderi-Meshkin and Setyaningsih, 2024).

### 3.1 Vascular tone and ECD

Endothelial cells play a crucial role in maintaining vascular tone by precisely regulating the secretion of various vasoactive substances, such as endothelium-derived relaxing factors (EDRFs) and endothelium-derived contracting factors (EDCFs), to maintain the dynamic balance of blood vessels. Among them, endogenous relaxing factors are represented by nitric oxide (NO), prostaglandin E2 (PGE2), prostaglandin I2 (PGI2), and endothelium-dependent hyperpolarizing factor (EDHF) (Vanhoutte, 1997; Kumar et al., 2020; Schmidt and de Wit, 2020). As the core vasodilator, NO is precisely catalyzed by endothelial nitric oxide synthase (eNOS) in endothelial cells during its synthesis, relying on the regulation of cofactors such as tetrahydrobiopterin (BH4), heme, flavin mononucleotide (FMN) and flavin adenine dinucleotide (FAD) (Förstermann and Sessa, 2012; Stuehr and Haque, 2019; Deng, 2021; Janaszak-Jasiecka et al., 2023). Once NO is generated, it is rapidly released into the bloodstream, binds to heme in VSMCs, activates soluble guanylate cyclase (sGC), triggers a series of signal transduction cascades, and ultimately achieves vasodilation and homeostasis maintenance by reducing intracellular Ca2+ concentration (Figure 3) (Francis et al., 2010; Montfort et al., 2017; Farah et al., 2018; Garcia and Sessa, 2019; Paulo et al., 2020). Meanwhile, PGE2 and PGI2 also activate adenylate cyclase by binding to specific receptors, increasing intracellular Cyclic adenosine monophosphate (cAMP) levels, and thus synergistically promoting smooth muscle relaxation (Chu et al., 2015). Additionally, the activation of calcium ion channels such as transient receptor potential vanilloid 4 (TRPV4) and chloride ion channels like TMEM16A in endothelial cells is also involved in the fine regulation of vasodilation (Heathcote et al., 2019; Ottolini et al., 2020; Mata-Daboin et al., 2023; Matsumoto et al., 2023; Garrud et al., 2024). In contrast, endothelin-1 (ET-1), as a potent vasoconstrictor, binds to its specific receptors to trigger downstream signaling pathways, resulting in smooth muscle cell contraction and vascular constriction (Banecki and Dora, 2023).

However, when ECD occurs, this delicate balance is disrupted. Abnormal expression and release of vasoactive factors have profound effects on the normal function of blood vessels (Wang et al., 2022). It is worth noting that these abnormal vasoactive factors not only damage vascular function but also act back on endothelial cells, exacerbating their injury and forming a vicious cycle. For example, reduced NO synthesis further exacerbates oxidative stress and inflammatory responses, leading to the deterioration of endothelial injury (Levine et al., 2012; Wu et al., 2021; Wang et al., 2022). This bidirectional regulatory mechanism provides a new perspective for understanding the pathophysiology of ECD and offers potential targets for developing new therapeutic strategies.

### 3.2 Oxidative stress, inflammation, and ECD

Oxidative stress arises from an imbalance between oxidative and antioxidant systems. This imbalance promotes the oxidation of LDL, reduces the bioavailability of NO, and enhances inflammatory responses, ultimately leading to endothelial dysfunction and potentially triggering the initiation and progression of AS (Kattoor et al., 2017; Akiyama and Ivanov, 2024; Loffredo and Carnevale, 2024). Ox-LDL can induce the upregulation of lectin-like ox-LDL receptor-1 (LOX-1) in endothelial cells. The increased expression of LOX-1 triggers endothelial cell apoptosis and inflammation, resulting in ECD (Gu et al., 2013; Mollace et al., 2015).

**FIGURE 4:**
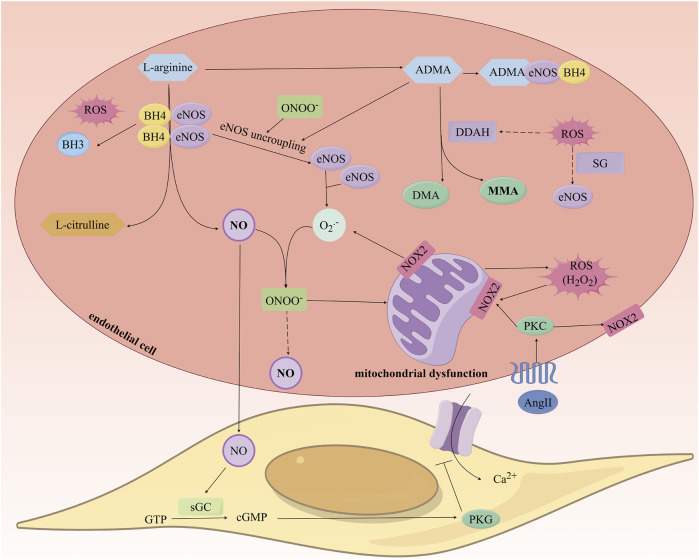
Study on the Mechanism of Nitric Oxide Production and the Impact of Oxidative Stress on Its Generation. ROS: Reactive oxygen species; eNOS: endothelial Nitric Oxide Synthase; NO: Nitric Oxide; ADMA: Asymmetric Dimethylarginine; DDAH: Dimethylarginine Dimethylaminohydrolase.

Reactive oxygen species (ROS) can directly interact with NO to form peroxynitrite (ONOO-), leading to the uncoupling of eNOS, increased levels of O2•-, and decreased synthesis of NO, thus forming a vicious cycle of NO consumption (Förstermann, 2010; Incalza et al., 2018). Additionally, ROS can downregulate the gene expression of eNOS and deactivate eNOS through S-glutathionylation, further reducing NO synthesis (Janaszak-Jasiecka et al., 2023). ROS also reduces the activity of dimethylarginine dimethylaminohydrolase (DDAH), leading to decreased asymmetric dimethylarginine (ADMA) degradation and upregulates protein arginine N-methyltransferase (PRMT) expression, which converts L-arginine into ADMA, resulting in elevated ADMA levels (Tsikas, 2020). ADMA, as an endogenous inhibitor of NOS, can also contribute to eNOS uncoupling and reduced NO levels (Bouras et al., 2013; Janes et al., 2019). Lastly, ROS oxidizes BH4 to dihydrobiopterin (BH3•), decreasing BH4 levels and leading to eNOS uncoupling, which effectively reduces the bioavailability of NO (Figure 4) (Yao and Abdel-Rahman, 2021; Janaszak-Jasiecka et al., 2023).

**FIGURE 5:**
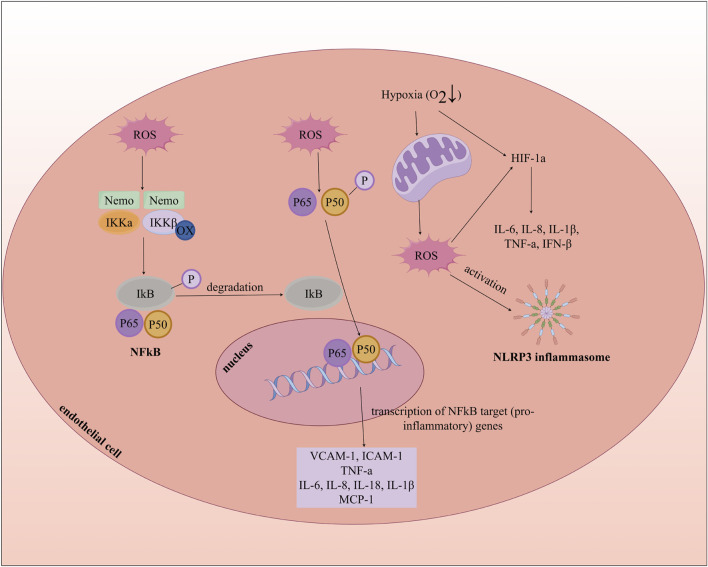
Oxidative Stress, Inflammation, and Endothelial cell dysfunction. MCP-1: Monocyte Chemoattractant Protein-1; INF-β: Interferon beta; NLRP3: nucleotide-binding domain (NBD), leucine-rich repeat (LRR), and pyrin domain (PYD)-containing protein 3.

Oxidative stress promotes inflammatory responses by activating nuclear factor-kappaB (NF-ĸB), hypoxia-inducible factor-1 alpha (HIF-1a), and the nucleotide-binding domain (NBD), leucine-rich repeat (LRR), and pyrin domain (PYD)-containing protein 3 (NLRP3) inflammasome, which leads to ECD and accelerates the progression of AS. NF-ĸB, a transcription factor that regulates the expression of inflammatory cytokines, is typically controlled in the cytoplasm by the inhibitory IĸB protein. Firstly, ROS can induce oxidation of the IĸB kinase (IKK) complex, causing phosphorylation of IĸB, resulting in its degradation and subsequent activation and nuclear translocation of NF-ĸB. The activation of this pathway results in the upregulation of adhesion molecules (such as VCAM-1 and ICAM-1), chemokines (such as MCP-1), and inflammatory mediators (such as IL-1β, IL-6, IL-8, IL-18, and TNF-a), creating a pro-inflammatory microenvironment that culminates in endothelial injury and intimal thickening (Sul and Ra, 2021; Shaito et al., 2022). Secondly, ROS can stabilize HIF-1a, which leads to the expression of pro-inflammatory genes associated with HIF-1a (Tian et al., 2021; Willson et al., 2022). Additionally, ROS activate the NLRP3 inflammasome complex, composed of NLRP3, the adaptor protein ASC, and caspase-1, where caspase-1 cleaves pro-IL-1β and pro-IL-18 into their mature forms. The release of IL-1β promotes the expression of adhesion molecules and chemokines, mediating the recruitment of leukocytes and monocytes in the early stages of AS (Figure 5) (Deng, 2021; Shaito et al., 2022). For instance, TNF-a leads to the generation of mitochondrial ROS (mtROS), activation of NADPH oxidase, and increased expression of inducible NOS (iNOS) in endothelial cells (Roca et al., 2022). Thus, oxidative stress, inflammation, and endothelial dysfunction are intricately intertwined processes.

### 3.3 Sex differences and ECD

Epidemiological studies indicate that although both men and women are susceptible to AS, men are more prone to developing ECD and AS when they are young. In contrast, women’s risk significantly increases post-menopause, potentially surpassing that of men at an advanced age (>85 years) (Martin et al., 2025).

According to the review by Robert et al., estrogen, particularly 17β-estradiol (E2), promotes vasodilation and improves endothelial function by enhancing the production of NO, H2S, and PGI2, while inhibiting the levels of ET-1. This process involves the binding of estrogen to estrogen receptor a (ERa)/ERβ and G Protein-Coupled Estrogen Receptor 1 (GPER-1) receptors. The ERa/ERβ receptors trigger the phosphorylation of eNOS via activation of the Src/the extracellular signal-regulated kinases 1 and 2 (ERK1/2)/phosphatidylinositol 3-kinase (PI3K)/Akt signaling pathway, whereas the GPER-1 receptor does so through the Src/epidermal growth factor receptor (EGFR)/PI3K signaling pathway, leading to the rapid release of NO for the regulation of vascular tension. Additionally, E2 binds to ERα/ERβ receptors, inducing the production of cyclic guanosine monophosphate (cGMP) via particulate guanylate cyclase-A (pGC-A), which then activates protein kinase G type Iβ (PKG-Iβ) to facilitate the rapid release of H2S from cystathionine G-lyase (CSE), thus promoting vasodilation. Moreover, estrogen enhances the sustained vasodilatory effect on the vascular system by upregulating the mRNA and protein expression of eNOS and H2S. Furthermore, E2 reduces the levels of prepro-ET-1, endothelin-converting enzyme (ECE), and ET-1 in the body (Robert, 2023). In addition to improving vasodilation, E2 can reduce inflammatory responses, improve ECD, and retard the development of AS by activating the AMP-activated protein kinase (AMPK)/peroxisome proliferator-activated receptor alpha (PPARα) signaling pathway (Robert, 2023).

The review further indicates that the primary source of circulating estrogen is E2 secreted by the ovaries in premenopausal women. However, in postmenopausal women and men, E2 levels drop significantly, mainly originating from adrenal secretion and the conversion of testosterone to E2 by aromatase in local tissues. During the same period, estrone (E1) becomes the main circulating estrogen, but its activity is only one-tenth that of E2. Consequently, the incidence of AS in postmenopausal women increases and may even surpass that in men (Robert, 2023).

## 4 Treatment of ECD in AS

### 4.1 Intervention of lifestyle factors

Adopting a healthy lifestyle plays a pivotal role in improving ECD. Poor lifestyle choices, such as unhealthy diets, lack of exercise, and smoking, can lead to endothelial damage, subsequently increasing the risk of AS (Naderi-Meshkin and Setyaningsih, 2024). To optimize endothelial function, we can start by adjusting our dietary structure. The 2019 ACC/AHA Guideline on the Primary Prevention of Cardiovascular Disease advocates a healthy dietary pattern based on plant-based diets and the Mediterranean diet. The core elements of this pattern emphasize increased consumption of fruits, nuts, fresh vegetables, legumes, whole grains, and fish (Arnett et al., 2019). The bioactive components of this dietary pattern have been proven to exhibit lipid-lowering, antioxidant, anti-inflammatory, and anti-thrombotic properties, all of which contribute to improved endothelial function (Shah et al., 2020; Yubero-Serrano et al., 2020). While optimizing the diet, it is necessary to strictly limit the intake of nutrients that have endothelial toxicity: 1) control sodium intake to <5 g/day to reduce Ang II-induced endothelial oxidative damage; 2) avoid trans fatty acids and processed meats (≤50 g per week) to reduce circulating levels of ox-LDL; 3) limit refined carbohydrates (energy intake ratio <10%) to maintain glycemic homeostasis. Through these meticulous adjustments, we can more comprehensively improve ECD and reduce the development of AS (Arnett et al., 2019; Huang et al., 2020).

The regulatory effect of exercise on endothelial cell function exhibits significant intensity dependence. Typically, exercise intensity is classified based on percentages of maximal oxygen uptake (VO_2max_), maximal heart rate (HR_max_), heart rate reserve (HRR), and one-repetition maximum (1RM) in resistance exercises. The specific classification is as follows: low intensity: <40% VO_2max_ or HRR <64% HR_max_, and 30%–50% 1RM; moderate intensity: 40%–60% VO2max or HRR at 64%–76% HR_max_, and 50%–70% 1RM; high intensity: >60% VO_2max_ or HRR >76% HR_max_, and 70%–85% 1RM. High-intensity exercise can be further divided into high-intensity continuous training and high-intensity interval training/high-intensity interval exercise (HIIT/HIIE). Additionally, according to different exercise frequencies, it can be categorized into acute exercise and long-term exercise (Li et al., 2023).

Long term low to moderate-intensity exercise enhances the expression of antioxidant enzymes such as superoxide dismutase (SOD) and glutathione peroxidase (GPX), while reducing the activity of oxidases including NADPH oxidase and xanthine oxidase, inhibiting expression of inflammatory cytokines, and enhancing the release of angiogenic factors such as vascular endothelial growth factor (VEGF), stromal cell-derived factor-1 (SDF-1), HIF-1, and matrix metalloproteinase-9 (MMP-9) (Wang et al., 2019; Li et al., 2023). Furthermore, long-term moderate-intensity exercise can upregulate the expression of mitochondrial inner membrane uncoupling protein 2 (UCP2) via the peroxisome proliferator-activated receptor gamma coactivator-1 alpha (PGC-1a)/PPARδ pathway, thereby reducing endoplasmic reticulum stress and lowering intracellular ROS levels (Zhou et al., 2016). Moderate-intensity aerobic or resistance exercise can increase the number of endothelial progenitor cells, promoting endothelial repair during vasculogenesis (Schlager et al., 2011). Additionally, moderate-intensity exercise and acute HIIT/HIIE can activate PI3K/Akt pathway, enhances the phosphorylation of eNOS, increases the release of NO, and decreases the production of ET-1, thus optimizing vascular tone (Wang et al., 2019). However, acute high-intensity continuous exercise may lead to eNOS uncoupling, resulting in reduced NO production and increased ET-1 generation (Boeno et al., 2019). Moreover, long-term high-intensity continuous exercise can cause excessive ROS production and elevate levels of inflammatory factors like IL-6, leading to an inflammatory response that further exacerbates endothelial dysfunction (Kayacan et al., 2021). Therefore, it is crucial to strictly control exercise intensity and frequency to avoid acute and long-term high-intensity continuous exercise.

On the other hand, smoking cessation is equally essential for improving endothelial function (Johnson et al., 2010). Cigarette smoke contains numerous and diverse ROS that have severe negative impacts on vascular endothelial function. Smoking causes a decrease in antioxidants such as ascorbic acid, cysteine, and methionine, further exacerbating endothelial damage (Ishida et al., 2024). Additionally, harmful components in cigarette smoke accelerate cellular aging processes, reducing cellular repair and regeneration capabilities (Zhou et al., 2023; Khan et al., 2024). The 2019 ACC/AHA Guideline on the Prevention of Cardiovascular Disease recommends the integration of dedicated tobacco treatment specialists within healthcare systems and the inclusion of tobacco use status as a vital sign, documented during every medical visit. The guideline also advocates that healthcare providers offer clear, firm, empathetic, and nonjudgmental cessation advice based on the specific situation of the patient. To further enhance the success rate of smoking cessation, the use of FDA-approved cessation medications, such as patches, gum, lozenges, nasal sprays, and oral inhalers, is recommended when necessary. Additionally, the implementation of smoke-free policies in enclosed spaces, public areas, and within 25 feet of ventilation intakes is emphasized to minimize the risk of exposure to secondhand smoke (Arnett et al., 2019).

In summary, through comprehensive lifestyle interventions such as a balanced diet, moderate exercise, and smoking cessation, we can potentially improve ECD to a certain extent, thereby slowing the progression of AS.

### 4.2 Pharmacological treatment

In recent years, research has discovered that certain drugs exhibit multiple effects. Consequently, drug therapies targeting ECD have become particularly important for the early prevention and treatment of cardiovascular diseases such as AS.

#### 4.2.1 Lipid-lowering drugs

According to the 2018 Cholesterol Clinical Practice Guidelines, for patients with an estimated 10-year atherosclerotic cardiovascular disease (ASCVD) risk of 5%–7.5%, it is recommended to use moderate-intensity statin therapy if other risk factors are present. For those with a 10-year ASCVD risk between 7.5% and 20%, using moderate-intensity statins is advised to achieve at least a 30% reduction in low-density LDL levels. For patients with a 10-year ASCVD risk of 20% or more, a more aggressive treatment strategy is suggested to reduce LDL levels by at least 50% (Arnett et al., 2019; Grundy et al., 2019). Statins are the first-line treatment for high cholesterol, working by inhibiting the activity of hydroxymethylglutaryl-coenzyme A reductase, which reduces endogenous cholesterol synthesis and subsequently lowers LDL levels in the blood. However, these drugs are not limited to lipid regulation; they also possess multiple functions in improving endothelial cell function in AS (Oesterle et al., 2017; Xu et al., 2021). Specifically, statins can enhance the phosphorylation of eNOS by activating the PI3K/Akt signaling pathway, which increases the production of NO and subsequently improves vasodilation (Kureishi et al., 2000; Xu et al., 2021). Additionally, research has shown that statins can enhance eNOS activity by decreasing the expression of the membrane protein caveolin-1, thereby disrupting its binding with endothelial eNOS in caveolae (Suh et al., 2010). Furthermore, statins promote polyadenylation levels of eNOS mRNA and increase its stability through Rho-mediated actin cytoskeleton remodeling (Tousoulis et al., 2014). In endothelial cells, statins effectively upregulate Krüppel-like factor 2 (KLF2) by inhibiting protein geranylgeranylation and activating the myocyte enhancer factor 2 (MEF2) pathway. This upregulation regulates eNOS transcription and significantly improves endothelial function (Sen-Banerjee et al., 2005). Moreover, these drugs inhibit intracellular Wnt/β-catenin signaling pathways, suppress endoplasmic reticulum stress, block the NF-ĸB signaling pathway, and upregulate the expression of the long non-coding RNA MANTIS, thereby exerting anti-inflammatory effects (Leisegang et al., 2019; Geng et al., 2020). In addition, statins enhance endothelial function by exhibiting anti-apoptotic properties and preventing Endothelial-to-Mesenchymal Transition (EndoMT) (Lai et al., 2018). These mechanisms suggest that statins not only excel in lipid-lowering but also show significant potential for targeted therapy of AS-related ECD.

However, while benefiting from these medications, we must remain vigilant about their potential adverse effects. Although statins are generally well-tolerated, they can still lead to abnormal liver function, an increased risk of new-onset diabetes, and side effects such as muscle pain, muscle damage, or even rhabdomyolysis (Ward et al., 2019; Laakso and Fernandes Silva, 2023). Therefore, in clinical practice, balancing the therapeutic efficacy of statins against their potential risks is a critical issue that requires careful consideration. Moving forward, we should explore more optimized dosing strategies to maximize efficacy and minimize side effects, or develop novel drugs to further enhance the treatment outcomes for ECD.

#### 4.2.2 Antihypertensive drugs

Based on the 2017 Hypertension Clinical Practice Guidelines, for adults with an estimated 10-year ASCVD risk of 10% or higher and an average blood pressure level of 130/80 mmHg or above, it is recommended to use antihypertensive medication for primary prevention of cardiovascular disease. For individuals with an estimated 10-year ASCVD risk of less than 10%, if their average blood pressure is 140/90 mmHg or above, initiating antihypertensive medication is also advised (Whelton et al., 2018; Arnett et al., 2019). Recent studies have demonstrated that many antihypertensive drugs have the potential to improve endothelial function while controlling blood pressure. Among these medications, renin-angiotensin system (RAS) inhibitors reduce angiotensin II (AngII) synthesis or block its AT II receptors, increase the stability of eNOS mRNA, enhance eNOS phosphorylation, decrease eNOS uncoupling, reduce the expression of NADPH oxidase (NOX), elevate BH4 levels, and inhibit bradykinin degradation. These actions collectively result in increased production of NO, thereby improving vascular endothelial relaxation function (Xu et al., 2021). Additionally, RAS inhibitors exhibit strong antioxidant properties by reducing the generation of ROS(Pastacı Özsobacı et al., 2024). It is noteworthy that AngII can induce fibroblast-specific protein 1 (FSP1) and alpha-smooth muscle actin (a-SMA) to trigger EndoMT and increase the expression of cell adhesion molecules such as P-selectin, VCAM-1, and ICAM-1; however, the RAS inhibitors can effectively reverse the EndoMT process and showcase significant anti-inflammatory effects (Tang et al., 2010; Murdoch et al., 2014).

In the targeted therapy of arterial AS-related ECD by antihypertensive drugs, β-blockers have drawn attention for their multiple mechanisms. Firstly, these drugs not only promote the release of NO, thereby improving vasodilation, but also significantly reduce the levels of fibrinogen, plasminogen activator inhibitor-1 (PAI-1), and homocysteine in patients with primary hypertension (Teger-Nilsson et al., 1991). At the same time, non-selective β-blockers with a-receptor antagonistic properties, such as carvedilol, contribute to improving endothelial cell function through their antioxidative characteristics (Haas et al., 2016).

Furthermore, calcium channel blockers (CCBs) exert effects by modulating endothelium-dependent relaxation responses and decreasing ET-1 production (Godfraind, 2000). These drugs not only provide hydrogen elements to combat free radicals through their aromatic ring structure, inhibit the oxidation of LDL, but also lower the expression of inflammatory factors such as C-reactive protein (CRP) and IL-6, demonstrating outstanding anti-inflammatory capabilities (Qi et al., 2017). Additionally, CCBs can effectively inhibit the action of PDGF, thus preventing excessive adhesion and aggregation of platelets (Soe et al., 2009).

However, it is important to note that while these medications demonstrate outstanding performance in improving endothelial function and preventing AS, their potential side effects cannot be overlooked. RAS inhibitors may lead to electrolyte imbalances, β-blockers may cause bradycardia and fatigue, and CCBs sometimes result in ankle edema or headaches (Graudins et al., 2016; Rirash et al., 2017; Salik et al., 2022). These dual impacts remind us of the necessity to carefully weigh the pros and cons in treatment strategies. Future research should focus on optimizing drug combinations to reduce the incidence of side effects, ultimately achieving more precise and personalized therapeutic approaches.

#### 4.2.3 Antidiabetic drugs

As we explore targeted therapeutic strategies for ECD in AS, antidiabetic medications have garnered increasing attention as a potential treatment modality. Not only do they aid in blood glucose control, but they also hold promise in slowing down the progression of AS by improving endothelial function. The table below summarizes the mechanisms of action and potential side effects of several commonly used antidiabetic medications in improving endothelial cell function (Table 1).

**TABLE 1:** Antidiabetic drugs used for improving endothelial cell dysfunction (ECD).

Drugs	Mechanism	Disadvantages
Insulin	Promotes the release of NO through the PI3K/Akt pathway, thereby improving vascular endothelial function (Xu et al., 2021)	(1) Benefits may be diminished in insulin resistance, impeding insulin action (Yudhani et al., 2023) (2) Long-term injection at the same site may cause subcutaneous fat atrophy or hyperplasia, and local reactions such as redness, swelling, and pain at the injection site
Metformin	(1) Inhibits the activation of NF-ĸB by activating AMPK and inhibiting the PI3K/Akt pathway, thereby suppressing inflammatory responses (Xu et al., 2021) (2) Regulates the survival, proliferation, and senescence of endothelial cells via the SIRT1 and LKB1/AMPK pathways (Ala and Ala, 2021) (3) Exhibits antioxidant effects, enhances autophagy flux, and reduces lipid accumulation (Kim et al., 2020a)	(1) Gastrointestinal side effects: nausea, vomiting, diarrhea, etc (McCreight et al., 2016) (2) Vitamin B12 deficiency, potentially leading to malnutrition and neurological disorders (Infante et al., 2021) (3) In patients with impaired renal function or severe hypoxia, lactic acidosis may occur (Mariano and Biancone, 2021)
SGLT2i	(1) Promotes NO production, contributing to endothelial function recovery (Xu et al., 2021) (2) Reduces oxidative stress and inflammatory responses (Xu et al., 2021) (3) Maintains the structural integrity of the glycocalyx (Xu et al., 2021) (4) Delays the senescence of endothelial cells (Tai et al., 2023)	(1) Urogenital infections: pyelonephritis, cystitis, etc. (Garofalo et al., 2019) (2) Blood volume-related reactions: symptomatic hypotension, dizziness, dehydration (Garofalo et al., 2019)
GLP-1R	(1) Promotes the production of NO, enhancing endothelial cell function (Xie et al., 2022) (2) Activates KLF2, preventing ox-LDL-induced monocyte adhesion and changes in endothelial permeability, protecting the integrity of the endothelial barrier (Chang et al., 2019) (3) Reduces ROS production and enhances the expression of endoplasmic reticulum oxidoreductase (ERO1α) in endothelial cells, aiding in the proper folding of endoplasmic reticulum proteins and thus alleviating endoplasmic reticulum oxidative stress (Helmstädter et al., 2020; Park et al., 2024) (4) Activates SIRT1 to prevent activation of NLRP3 inflammasome, exerting anti-inflammatory effects (Luo et al., 2019) (5) Increases the expression of endothelial ATP-binding cassette A1 and promotes cholesterol efflux, reducing lipid accumulation (Yin et al., 2016)	May increase the risk of hypoglycemia and the incidence of pancreatic and thyroid cancer (Zhao et al., 2021)
DPP-4i	(1) Reduces blood glucose levels by blocking the degradation of GLP-1 and glucose-dependent insulinotropic polypeptide (GIP), thereby improving endothelial cell function through GLP-1-dependent pathways (Xu et al., 2021) (2) Activates AMPK to promote metabolic activity and antioxidant capacity of endothelial cells, reducing inflammatory responses (Qi et al., 2019) (3) Inhibits activator protein 1 (AP-1) and NF-ĸB, thereby reducing inflammatory responses (Ma et al., 2019) (4) Activates SIRT1 to promote anti-aging and anti-inflammatory effects in endothelial cells (Jiang et al., 2019) (5) Activates the stromal cell-derived factor-1/chemokine receptor type 4 (SDF1/CXCR4) axis, increasing endothelial regeneration after vascular injury (Ma et al., 2019)	Risk of increased incidence of cancers (Niazmand et al., 2024)

#### 4.2.4 Antiplatelet drugs

The 2019 ACC/AHA Cardiovascular Disease Prevention Guidelines suggest that for adults aged 40 to 70 with a higher risk of ASCVD and without an increased risk of bleeding, a daily oral intake of 75–100 mg low-dose aspirin could be considered for primary prevention. However, for adults over the age of 70 or individuals of any age with an increased risk of bleeding, low-dose aspirin should not be used as a means of primary prevention for ASCVD (Arnett et al., 2019). Aspirin, as a classic antiplatelet agent, significantly reduces the production of thromboxane A2 (TXA2) within platelets by irreversibly inhibiting the activity of cyclooxygenase-1 (COX-1), thereby effectively inhibiting platelet aggregation. This mechanism plays a key role in preventing the formation of atherosclerotic plaques (Badimon et al., 2021). Similarly, drugs such as clopidogrel and ticagrelor successfully block the adenosine diphosphate (ADP)-mediated platelet aggregation process by inhibiting the P2Y12 ADP receptor, thereby helping to maintain the normal function of endothelial cells (Scanavini-Filho et al., 2022). Furthermore, GPIIbIIIa inhibitors, by binding to activated GPIIbIIIa platelet receptors, prevent the binding of platelets to fibrinogen, thus inhibiting platelet cross-linking and thrombosis, and further protecting arterial endothelial cells from damage caused by ischemia and hypoxia (Liu et al., 2023).

Research has confirmed that these antiplatelet drugs play a crucial role in preventing and improving ECD. In particular, aspirin can also increase eNOS and cGMP levels, inducing the release of NO from vascular endothelium and further improving vascular ECD (Ou et al., 2012). Additionally, antiplatelet drugs also have anti-inflammatory effects on endothelial cells. For example, Ikonomidis et al. found that daily intake of 300 mg aspirin significantly reduced the expression of inflammatory biomarkers such as IL-6, MCSF, and CRP (Ikonomidis et al., 1999). After activation of the P2Y12 receptor, the released alpha granules contain platelet P-selectin and various soluble pro-inflammatory factors. P-selectin can bind to PSGL-1 on monocytes, activating the NF-ĸB signaling pathway and promoting the release of inflammatory factors such as TNF-α, IL-1β, IL-8, and MCP-1. Additionally, during this process, the induced appearance of GPIIb/IIIa interacts with CD11b through fibrinogen bridging, further enhancing platelet-leukocyte aggregation. Based on these mechanisms, P2Y12 receptor inhibitors like clopidogrel can effectively mitigate the inflammatory response (Parker and Storey, 2024). In conclusion, through the integration of multiple mechanisms, antiplatelet drugs provide robust support for targeted therapy of ECD associated with AS.

However, these drugs also have certain limitations and side effects in clinical application, such as increased risk of bleeding and adverse gastrointestinal reactions (Sostres et al., 2019). Therefore, future research can focus on developing safer and more effective antiplatelet drugs or treatment strategies, exploring the combined application of antiplatelet drugs with other drugs, and utilizing advanced technologies such as genomics to achieve precise treatment for patients. Looking ahead, with continued efforts, we hope to find better approaches to balance the therapeutic effects and side effects of antiplatelet drugs, providing more effective means for treating ECD associated with AS.

### 4.3 Novel therapeutic approaches

In recent years, with the continuous advancement of science and technology, numerous novel approaches have emerged in the field of targeted therapy for ECD in AS. Among them, nanomedicine, stem cell therapy, and genetic therapy have attracted significant attention, offering new possibilities and hope for the treatment of this disease.

#### 4.3.1 Nanomedicine

Numerous studies have demonstrated that by precisely regulating the physical properties of nanomaterials and combining them with specific targeting delivery systems, drugs can be effectively delivered to endothelial cells. The advantage of this approach is that it significantly improves the bioavailability of drugs while reducing related side effects, opening up new pathways for improving endothelial cell function (Taneja et al., 2019; Cheng J. et al., 2023). For example, amphiphilic polysiloxane nanoparticles can promote the release of NO through endocytosis via caveolae in human aortic endothelial cells (HAECs), thus improving endothelial function (Nishikawa et al., 2009). Additionally, Annapoorna Mohandas et al. successfully developed nanocurcumin and arginine-incorporated chitosan hydrogel, which demonstrated the ability to enhance the phosphorylation of eNOS in endothelial cells *in vitro*, along with antioxidant activity, effectively preventing hypoxia-induced endothelial injury (Mohandas and Rangasamy, 2021). Another noteworthy study conducted by Xiao et al., who found that in apolipoprotein E-deficient mice fed a high-fat diet, oral administration of CS SeNPs and Na2SeO3 (40 μg Se/kg/day) for 10 weeks significantly increased NO levels and decreased the expression of adhesion molecules, thereby alleviating vascular ECD and reducing atherosclerotic lesions (Xiao et al., 2021).

Despite the immense potential of nanomaterials in targeted vascular endothelial therapy for AS, there are still numerous challenges during clinical translation. Among these, issues such as variations in artery diameter, continuous blood flow shear stress, and blood marginalization require careful consideration. Furthermore, the safety, stability, reproducibility of nanomaterials, as well as regulatory and ethical requirements, are also significant factors that cannot be ignored (Taneja et al., 2019). It is worth noting that some nanomaterials may have adverse effects on endothelial cells. For instance, silica nanoparticles (Nano-SiO2) significantly reduce NO levels and eNOS activity by activating the PI3K/Akt/mTOR signaling pathway, increase inflammatory responses, activate autophagy, and ultimately lead to ECD (Duan et al., 2014). Moreover, iron oxide nanoparticles (Fe2O3 and Fe3O4) may damage HAECs and enhance the adhesion of monocytes to these cells, thereby promoting the development of AS (Zhu et al., 2011). Other studies have found that exposure to silver nanoparticles (AgNPs) may inhibit the proliferation of human umbilical vein endothelial cells (HUVECs), activate the IKK/NF-ĸB signaling pathway, and trigger oxidative stress and inflammatory responses, which are potential risk factors for early AS (Shi et al., 2014). Therefore, in future research, we need to continue optimizing the biocompatibility and safety of nanomaterials to ensure their optimal efficacy in the field of targeted therapy for ECD in AS.

#### 4.3.2 Stem cell therapy

Mesenchymal stem cells (MSCs) have opened up new perspectives and research directions for the treatment of ECD due to their unique advantages, such as low immunogenicity, strong self-renewal capacity, ease of culture and rapid expansion *in vitro*, as well as the potential to differentiate into multiple cell lineages under appropriate conditions. Studies have demonstrated that MSCs can promote angiogenesis and accelerate the repair and regeneration of endothelial cells (Li et al., 2017). Furthermore, MSCs can upregulate the expression levels of eNOS, argininosuccinate synthase 1 (ASS1), KLF2 and KLF4, while reducing the levels of blood lipids, lactate dehydrogenase (LDH), and von Willebrand factor (vWF). These molecules play a critical role in maintaining endothelial cell function. Specifically, eNOS improves vascular dilation by catalyzing the production of NO from L-arginine. ASS1 is a urea cycle enzyme widely present in endothelial cells that catalyzes the condensation of citrulline and aspartate to form argininosuccinate, which is then regenerated into arginine (Cao et al., 2024). Studies have shown that MSCs significantly enhance ASS1 expression, promoting arginine regeneration and maintaining adequate substrate levels for NO production (He et al., 2020). KLF2 exerts anti-inflammatory effects by inducing Smad7 expression and reducing activating protein-1 (AP-1) activity to inhibit the TGF-β signaling pathway. In contrast, KLF4 attenuates TGF-β activity by upregulating the expression of extracellular matrix protein Tenascin-X (TN-X) in endothelial cells and prevents EndoMT. Together, they complement each other in forming an essential barrier that protects endothelial cells from inflammatory and fibrotic damage (Kotlyarov and Kotlyarova, 2023). Of particular note, MSCs possess the capability to alleviate oxidative stress damage and secrete anti-inflammatory cytokines like IL-10 and interleukin-1 receptor antagonist (IL-1RA) to suppress local inflammatory responses (Liu et al., 2022). Additionally, MSCs release various growth factors, including basic fibroblast growth factor (bFGF), insulin-like growth factor-1 (IGF-1), hepatocyte growth factor (HGF), and VEGF, to promote cell proliferation and stabilize vulnerable plaques in AS (Yamahara et al., 2014). These findings provide new possibilities and research directions for targeted therapy of ECD associated with AS (Li et al., 2017).

However, despite the tremendous therapeutic potential of MSCs, many challenges and issues remain in practical applications. Firstly, obtaining sufficient quantities and quality of stem cells is an urgent problem to be solved. Currently, the source, isolation, expansion, and pretreatment processes of stem cells have not been standardized, which may affect the final effect of cell therapy (Torre et al., 2015). Secondly, the optimal route of administration, dosage, and timing of stem cell therapy still need further exploration. In addition, the long-term safety and effectiveness of stem cell therapy need to be verified through large-scale, long-term clinical trials (Sun et al., 2016). In summary, MSCs therapy for atherosclerotic ECD has broad prospects, but continuous exploration and improvement are still necessary.

#### 4.3.3 Genetic therapy

Genetic therapy, as a cutting-edge and highly precise therapeutic approach, is gradually unveiling its vast potential. Thanks to the rapid advancements in bioinformatics technology and machine learning, we have been able to not only deeply explore the key Micro RNAs (miRNAs) (Table 2) regulating endothelial cell function and a series of differentially expressed genes (DEGs) (Table 3) that regulate endothelial cell function, achieving a breakthrough from “molecular target identification” to “cellular function remodeling.” For instance, Laena et al. successfully reprogrammed some endothelial cells into an endothelial-like state by overexpressing Oct-3/4, Sox-2, and Klf-4 (OSK) transcription factors in these cells. This precise transcriptional regulation strategy effectively reversed the senescent phenotype of endothelial cells and inhibited pro-atherosclerotic EndoMT (Pernomian et al., 2024). This multifaceted intervention approach, which encompasses both omics-based molecular targeted therapy and transcriptional reprogramming for cellular function restoration, has not only significantly enhanced the efficiency of drug development, but also injected robust momentum into the thriving field of personalized medicine. More importantly, by employing a dual-path therapeutic approach that targets key molecular nodes in pathological processes while systematically remodeling endothelial cell homeostasis, we hold the promise of effectively halting the progression of AS, thereby opening up novel therapeutic pathways for patients.

**TABLE 2:** Micro RNAs (miRNAs) regulating endothelial cell function.

miRNAs	Endothelium	Mechanism
*miR-10a*	HAECs	Reduces expression of mitogen-activated kinase kinase kinase 7 (MAP3K7) and beta-transducin repeat-containing gene (betaTRC), inhibiting IkBa degradation, p65 nuclear translocation, and NF-ĸB activation. Lowers inflammatory biomarkers such as MCP-1, IL-6, IL-8, VCAM-1, and E-selectin (Fang et al., 2010)
*miR-17-3p*	HUVECs	Inhibits TNF-a-induced E-selectin expression and neutrophil adhesion to endothelial cells (Suárez et al., 2010)
*miR-21*	HUVECs	Targets regulation of DDAH-1-ADMA-eNOS-NO pathway promotes AS (Yang et al., 2020)
*miRNA-26a-5p*	MCAECs^a^, HUVECs	Activates PI3K/AKT pathway by targeting *PTEN*, reduces ET-1, TxA2, AngII expression, and increases eNOS and PGI2 expression (Jing et al., 2019)
*miR-31*	HUVECs	Inhibits TNF-a-induced ICAM-1 expression and neutrophil adhesion to endothelial cells (Suárez et al., 2010)
*miR-31-5p*	HUVECs, HAECs	Reduces eNOS mRNA stability, leading to eNOS downregulation and inhibition of NO production (Kim et al., 2018)
*miR-34a*	HUVECs	Promotes endothelial cell senescence by inhibiting *SIRT1*, and inhibit cell proliferation by suppressing the cell cycle process (Ito et al., 2010)
*miR-92a*	HUVECs	Targets KLF2, KLF4, and suppressor of cytokine signaling 5 (SOCS 5) expression (Wu et al., 2011)
*miR-101*	HUVECs	Regulates the activity of eNOS by inhibiting the expression of *EZH2* (Xu et al., 2018)
*miR-106b*	HAECs	Inhibits endothelial cell apoptosis through the *PTEN*/P13K/AKT signaling pathway (Zhang et al., 2020b)
*miR-125a/b-5p*	MHMECs^b^, MBMECs^c^	Inhibits ET-1 expression (Li et al., 2010)
*miR-126*	HUVECs	Inhibits TNF-a-induced VCAM-1 expression and leukocyte adhesion to endothelial cells (Harris et al., 2008)
*miR-130a*	MAECs^d^, HUVECs	Reduces the senescence of endothelial cells; enhance the bioavailability of NO(Dhahri et al., 2020)
*miR-133a*	HUVECs	Targets the reduction of GCH1 protein expression, leading to decreased BH4 protein expression and eNOS decoupling (Li et al., 2016)
*miR-155*	HUVECs	Reduces eNOS expression to decrease NO production (Sun et al., 2012a)
*miR-181b*	HUVECs	Targets and inhibits importin-a3 and a set of NF-ĸB-regulated genes, thereby inhibiting NF-ĸB signaling pathway activation and leukocyte adhesion to endothelial cells (Sun et al., 2012b)
*miR-199a-3p/5p*	BAECs^e^	Promotes eNOS activity and reduces its degradation by inhibiting *miR-199a-3p/5p*, thereby increasing the bioavailability of NO(Joris et al., 2018)
*miR-204*	HPAECs^f^	Inhibits hypoxia-induced EndoMT (Liu et al., 2019)
*miR-221*	HAECs	Inhibits eNOS activity (Rippe et al., 2012)
*miR-222*	HAECs	Inhibits eNOS activity (Rippe et al., 2012)
*miR-363-3p*	HUVECs	Targets regulation of KLF2 expression (Gao et al., 2020)
*miR-455*	HUVECs	Regulates TGF-β-induced EndoMT by targeting the expression of zinc finger E-box homeobox 1 (ZEB1) (Zhang et al., 2020a)
*miR-488-3p*	RAECs^g^	Blocks the TGF-β/Smad pathway to inhibit EndoMT (Guan et al., 2020)
*miR-652-3p*	HUVECs	Reduces ISL1 levels, activates eNOS, and promotes NO production (Liang et al., 2020)

^a^ MCAECs: Mouse coronary artery endothelial cells.

^b^ MHMECs: Mouse cardiac microvascular endothelial cells.

^c^ MBMECs: Mouse cerebrum microvessel endothelial cells.

^d^ MAECs: Mouse aortic endothelial cells.

^e^ BAECs: Bovine aortic endothelial cells.

^f^ HPAECs: Human pulmonary artery endothelial cells.

^g^ RAECs: Rat aortic endothelial cells.

**TABLE 3:** Differentially expressed genes (DEGs) regulating endothelial cell function.

Mechanism		Gene name
Endothelial cell senescence (Xiang et al., 2022)	Suppression	*MMP9, BCL6*
	Induction	*ETS2*
Endothelial cell death (Pinheiro-de-Sousa et al., 2023)	Suppression	*JUNB, SALL4, NOX4, VEGFA, FOSB*
	Induction	*NRA42, FOS, TRAF1, HGF, SLC7A5*
Mitochondrial dysfunction (Wang et al., 2024a)	Suppression	*SGCA*
	Induction	*GAL*
Cellular oxidative stress (Pinheiro-de-Sousa et al., 2023; Liang et al., 2025)	Suppression	*JUNB, HEY1, SOCS1, NR4A3, MAFG*
	Induction	*TRAF1, SLC7A5, FOS, HGF, NR4A2, OAS1*
Cellular inflammatory response (Ma et al., 2022; Pinheiro-de-Sousa et al., 2023; Wang et al., 2024a; Wang et al., 2024b; Liang et al., 2025)	Suppression	*CSF2, NR4A3, ST6GAL1, UPP1, VEGFA, TRAF1, SLC7A5, FOS, HGF, NR4A2*
	Induction	*IFI6, FSCN1, PHACTR1, HGF, FOS, SLC7A5, NR4A2, TRAF1, LDLR, OAS1, CCR1, NCKAP1L*
Reduction of NO production (Ma et al., 2022; Liang et al., 2025)		*PHACTR1, OAS1*
Induction of Genotoxic Stress (Sinitsky et al., 2022)		*MDM2*
Repair of early endothelial lipid peroxidation damage (Dou et al., 2019)		*CCL2*

By precisely regulating the expression of specific genes, a novel therapeutic approach has been pioneered to combat ECD in AS. However, significant technical challenges still lie ahead in improving the targeting accuracy and therapeutic efficiency of gene therapy. Ensuring that the treatment reaches the disease site directly and produces the desired effect is of utmost importance. Furthermore, safety issues cannot be overlooked, as any inadvertent manipulation may cause irreversible damage to the patient’s genome. Therefore, rigorous clinical trials and comprehensive safety assessments must be conducted before implementing gene therapy. Additionally, the current high costs of gene therapy technology and drug development undoubtedly hinder its widespread accessibility among patients (Gonçalves and Paiva, 2017; Ylä-Herttuala, 2019; eBioMedicine, 2024). Thus, exploring ways to reduce the costs of gene therapy to enhance its affordability and accessibility has become an urgent challenge to be tackled.

## 5 Conclusion

The development and improvement of therapies for ECD have emerged as a significant research area. Through comprehensive study and analysis of existing related research, we have noticed that pharmacological treatments not only reduce cardiovascular risk factors but also exhibit the ability to improve endothelial cell function. However, there are also obvious limitations, such as side effects and drug resistance issues. Nevertheless, despite the greater potential demonstrated theoretically by the promotion and application of emerging therapies, their clinical efficacy and safety still require substantial verification. Therefore, it is imperative for us to continually sharpen our perspectives and intellect in practice, seeking an optimal treatment paradigm that integrates traditional therapies with emerging ones, achieving the best balance between safety and effectiveness.
